# Impact of Body Mass Index on Static Postural Control in Adults With and Without Diabetes: A Cross-Sectional Study

**DOI:** 10.3389/fendo.2021.768185

**Published:** 2021-12-24

**Authors:** Lianhua Yin, Jiawei Qin, Yannan Chen, Jinjin Xie, Cuiping Hong, Jia Huang, Ying Xu, Zhizhen Liu, Jing Tao

**Affiliations:** ^1^ Health Management Center, The Second Affiliated Hospital of Fujian University of Traditional Chinese Medicine, Fuzhou, China; ^2^ College of Rehabilitation Medicine, Fujian University of Traditional Chinese Medicine, Fuzhou, China; ^3^ Department of Rehabilitation Medicine, Quanzhou First Hospital Affiliated to Fujian Medical University, Quanzhou, China; ^4^ Key Laboratory of Orthopedics and Traumatology of Traditional Chinese Medicine and Rehabilitation, Fujian University of Traditional Chinese Medicine, Ministry of Education, Fuzhou, China; ^5^ National-Local Joint Engineering Research Center of Rehabilitation Medicine Technology, Fujian University of Traditional Chinese Medicine, Fuzhou, China

**Keywords:** type 2 diabetes mellitus, healthy, BMI, Center of Pressure (CoP), static postural control

## Abstract

**Aim:**

The objective of this research was to determine the static postural control differences measured from a force platform in Type 2 diabetes mellitus (T2DM) and healthy control groups with different levels of body mass index (BMI), and detect the static postural control difference between T2DM and healthy control groups stratified by different BMI category. This research also explored the relationship of BMI and static postural performance.

**Methods:**

We recruited 706 participants with T2DM and 692 healthy controls who were sufficiently matched for age, gender, and BMI in this cross-sectional study. The participants were stratified into three groups by BMI: normal weight, overweight, and obesity. All participants performed two-legged static stance postural control assessment on a firm force platform. The Center of Pressure (CoP) parameters were collected under eyes-open and eyes-closed conditions. Mann–Whitney U test was used to compare the static postural control parameters within each BMI category in both groups. The static postural control parameters among different weight groups were compared by Kruskal–Wallis test, *post hoc* pair-wise comparison were conducted. Generalized linear model was conducted to examine the association between BMI and static postural control parameters while controlling for confounding factors.

**Results:**

Healthy control group had statistical difference in most CoP parameters compared to T2DM group based on all BMI categories. Normal weight participants presented significant difference compared with overweight and/or obesity for total track length (TTL) and velocity of CoP displacements in Y direction (V-Y) under eyes-open condition, and for most CoP parameters under eyes-closed condition in both groups. There were statistically significant correlations between BMI and most static postural control parameters under only eyes-closed condition according to the result of generalized linear model.

**Conclusion:**

T2DM patients had impaired static postural control performance compared to healthy controls at all BMI categories. The findings also indicated the association between BMI and static postural control, where higher BMI individuals showed more static postural instability in both T2DM and healthy controls.

## Introduction

Postural balance is an important foundation of standing, walking, and activities of life. Maintaining static upright stance involves somatosensory inputs, central nervous system integration, and automatic postural response outputs (1). Falls in the elderly are the main causes of fatal injury, and the most common reason of non-fatal injury related to hospitalization (2). The report from the Nation Council on Aging showed that more than 25% elderly aged over 65 fall each year, and an average of 1 elderly death caused directly or indirectly by the falls every 19 min (2). A systematic review found the hospitalization costs for fall-related injury in Chinese elderly were $1,768 (3). The falls of the elderly bring serious negative influence in aspects of economy, function, and psychology for the elderly. Postural control impairments were considered as a primary risk factor for falling (4).

There are approximately 451 million persons diagnosed as diabetic over the world from the survey of the International Diabetes Federation, and most of these patients were Type 2 Diabetes Mellitus (T2DM) (5). It was reported that the incidence of falls for T2DM individuals was 39%, which was much higher than those without T2DM (6). Traditionally, most studies evaluated postural control based on clinical tests, such as single leg stance test, time up and go test, and Berg balance scale. These clinical tests were insufficiently sensitive to assess postural control and screen for fall risk (7). Recently, laboratory tests that collect CoP variables from a force plate-form had better identification of future falls and quantitative assessment of postural control (8). Quiet standing posturography was an appropriate tool for the fall risk and postural control evaluation. Some previous studies revealed that T2DM had poorer postural control performance in aspects of limit of stability and pressure center displacement variables compared to healthy controls (9, 10). A cross-sectional study measuring postural instability by static posturography found that T2DM individuals had a higher number of falls within the preceding years compared to healthy controls, and the main factor associated with falls was increased postural instability (11). However, another research found no significant difference between T2DM and healthy controls in terms of Centre of Pressure (CoP) variables (12).

Many factors are associated with higher fall risk; T2DM was one of the major risks (2). In addition to polypharmacy and age, diabetes-related muscle strength loss, sensory perception decrease, peripheral neuropathy, and decline of cognitive function would lead to greater imbalance (2). Besides, body mass index (BMI) had a strong positive relationship with postural instability (13). Obese and overweight individuals showed decreased function and postural stability compared with normal weight individuals (14). Previous studies showed that obese elderly had poorer performance in the sensory organization test, limits of stability, and time up and go test (15). Additionally, it also showed that obese elderly had a higher risk of falls compared to non-obese elderly (15). T2DM patients also showed postural control deficit with increasing BMI (10, 16). Teasdale (17) observed that measurements of postural stability were improved in obese subjects after losing weight, a strong linear relationship between weight loss magnitude and postural control improvement, suggesting that weight played an important role in postural stability. Despite previous work in this area, the knowledge of BMI impacts on postural control in individuals with or without T2DM is still limited (18). Although the research observed a relationship of poorer postural control and higher BMI, the role of T2DM in this association is not well established. Meanwhile, weight management is a crucial issue in T2DM, and the reduction of BMI was associated with better metabolic control for patients with T2DM (19, 20). Previous research had not dealt with postural balance comparison between T2DM and healthy controls under different BMI categories. The decline of postural control is one of the major factors contributing to a higher fall risk (21); prevention of falls by monitoring postural control could be an important public health issue. Identifying the general relationship between BMI and postural control was a critical step for T2DM individuals.

The objective of this research was to determine the static postural control differences measured from a force platform in T2DM and healthy control groups with different levels of BMI, and detect the static postural control difference between T2DM and healthy control groups stratified by different BMI categories. This research also explored the relationship of BMI and static postural control performance.

## Methods

### Participants

We recruited 706 participants with T2DM in this cross-sectional study, and the other 692 non-T2DM participants were sufficiently matched for age, gender, and BMI. All the participants in this research were recruited by contacting the the physical examination center of the Second Affiliated Hospital of Fujian University of Traditional Chinese Medicine between January 2015 and June 2020. Interested individuals were encouraged to contact the investigator, then completed relevant questionnaire that was designed to verify their admissibility in this research. This questionnaire contained an introduction of this research, inclusion and exclusion criteria, and an evaluation process of postural control.

For the observation group (T2DM group), the inclusion criteria were: voluntary to participate this study, age from 55 to 75, being diagnosed with T2DM by physician and subjective to use of metformin or insulin. The exclusion criteria contained blind or deaf participants, symptoms of osteoarthritis, pain in lower limbs or spine, rheumatic disease, severe cognitive impairment, malignant tumor, neuromuscular disease that affecting muscle strength or balance function, cardiovascular disorders (including stroke), surgical intervention for spine or lower limbs, knee or ankle injury in last 12 months.

This study was approved by the Second Affiliated Hospital of Fujian University of Traditional Chinese Medicine Ethics Committee (approval number SPHFJP-K2019059-02).

### Assessment

Anthropometric information and clinical materials, namely, age, sex, weight, height, blood pressure, blood glucose, and current smoking status were collected during the recruitment process.

According to the Chinese BMI category established by the World Health Organization (22), all participants were divided into normal weight group (BMI = 18.5–23.9 kg/m^2^), overweight group (BMI = 24–27.9 kg/m^2^), and obese group (BMI >28 kg/m^2^). Underweight participants whose BMI was less than 18.5 kg/m^2^ were excluded to avoid bias results since the sample size of those underweight was too small.

A well-trained physiotherapist measured the static postural control on a force platform (Super Balance, Bismarck, Germany). The evaluation was conducted in a quiet and bright indoor environment. The participants took off their shoes, stood on the force platform (hard platform) with their arms on the sides and feet hip width. The participants kept their body upright and the Center of Pressure (CoP) stable as far as possible, the test steps including: stood on the platform with eyes open for 30 s at first, and then stood with eyes closed for 30 s after a rest of 1 min. All participants were evaluated in this order, and only perform once each trial. The participants looked straight ahead in eye-open condition, and closed their eyes voluntary in eyes-closed condition. All sessions for all participants were conducted in the morning. The change track of body pressure center (posture diagram) was recorded under eyes-open and eyes-closed conditions. The evaluation would be stopped and repeated if the participants shook, turned around, took a step, or held the handrails. The evaluator stood next to the participants to protect them.

The following parameters were derived (21, 23). Total track length (TTL, mm), where the TTL refers to the road of CoP passed in a certain period of time. The total length of the line reflects the degree of spontaneous body sway. The sway area (SA, mm^2^), where the SA refers to the size of CoP tracking map. The whole postural balance could be judged by SA, which is inversely proportional to the ability to maintain balance and stability. Track length per unit area (TTL/SA, mm), where the ratio of the total track length to the area of the track map value, also reflects the participant’s postural control ability. Maximum sway length of CoP along X direction (MSL-X, mm) and Y direction (MSL-Y, mm), which refers to the maximum sway distance in medial-lateral and antero-posterior directions. The velocity of CoP displacements in X (V-X, mm/s) and Y (V-Y, mm/s) directions, refers to the velocity of CoP sway in medial-lateral and antero-posterior directions. Romberg quotient (RQ), refers to the area ratio tracking map area ratio under the condition of eyes-closed and eyes-open in upright position. It reflects the compensation ability of vestibular and proprioception when patients maintain their posture without visual factors. RQ reflects the impact of visual feedback on static postural control.

### Statistical Analysis

All data were analyzed with Statistical Package for the Social Sciences (SPSS, v.24). The Kolmogorov–Smirnov test was used to verify if the variables were normally distributed. All normally distributed continuous data were presented as means and standard deviation, non-normally distributed continuous data were presented as median (P25, P75), and categorical data were presented as frequencies or percentages. Two-sample t-test or Mann–Whitney U test was used to compare the demographic data between the T2DM group and the healthy control group. The postural stability parameters among different BMI levels for both groups were compared by analysis of variance (ANOVA) or Kruskal–Wallis test, *post hoc* pair-wise comparison were conducted. Chi-square analysis was conducted for categorial data. Generalized linear model was conducted to examine the association between BMI and postural stability parameters while controlling for heart rate, hypertension status, and fasting blood glucose. *P <*0.05 was identified as statistical significance.

## Results

**Table 1** presents the characteristic of participants. There were no significant difference in age, gender, height, weight, BMI, BMI category, and current smoking status between the T2DM and the HC groups (all *P >*0.05). The hypertension portion and heart rate were higher in the T2DM group (both *P <*0.05). As expected, significant difference exists for FBG between the two groups.

**Table 1:** Characteristic of the samples.

	T2DM (n = 706)	HC (n = 692)	*P*
Age (years)	64 (57, 71)	65 (58, 71)	0.128
Gender (male/female)	454/252	410/282	0.052
Height (m)	1.65 (1.58, 1.70)	1.64 (1.57, 1.69)	0.116
Weight (kg)	65.5 (59.5, 72.3)	64.7 (58.3, 71)	0.051
BMI (kg/m^2^)	24.38 (22.55, 26.27)	24.32 (22.42, 26.33)	0.671
BMI category			0.799
1 Normal weight	308 (43.6%)	314 (45.4%)	
2 Overweight	310 (43.9%)	293 (42.3%)	
3 Obesity	88 (12.5%)	85 (12.3%)	
Systolic pressure (mmHg)	136 (125, 147)	133 (121, 144)	0.004
Diastolic pressure(mmHg)	79 (72, 86)	79 (72, 86.5)	0.532
Hypertension n(%)	394 (55.8%)	278 (40.20%)	0.001
Current smoking (yes)	91 (12.90%)	69 (10.00%)	0.087
Heart rate (bpm)	76 (68, 82)	74 (67, 81)	0.001
FBG(fasting blood glucose)	6.99 (6.24 8.42)	5.39 (5.03, 5.85)	0.001

T2DM, type 2 diabetes mellitus; HC, healthy control; BMI, body mass index.

**Table 2** provided a description of static postural control parameters stratified by BMI in the T2DM and the HC groups. Regarding the static postural control parameters under eyes-open condition, the statistical analysis results indicated that the T2DM group performed poorly than the HC groups for TTL, SA, MSL-X, MSL-Y, V-X, and V-Y in all three groups based on BMI category (normal weight, overweight, and obese). For T2DM group, we found significant difference for TTL and V-Y in normal weight participants compared with overweight/obese participants. For the HC group, we also found significant difference for TTL and V-Y in normal weight participants compared with overweight/obese participants. Normal weight participants in the HC group presented significant difference for MSL-Y compared with obese participants.

**Table 2:** Comparison of static postural control parameters according to both groups and all BMI categories.

		Groups	BMI category				*Post hoc* pair-wise comparison (*P*)		
			Normal weight	Overweight	Obesity	*P*	1 vs 2	1vs3	2vs3
Eyes-open	TTL	T2DM	184.74 (145.35, 245.85)	208.49 (167.32, 267.13)	221.09 (166.61, 277.44)	0.002	0.004	0.031	1
		HC	138.17 (114.3, 170.6)	149.39 (120.06, 186.44)	149.03 (123.47, 207.07)	0.002	0.006	0.04	1
		P	<0.001	<0.001	<0.001				
	SA	T2DM	123.6 (75.98, 203.86)	147.57 (93.22, 213.94)	132.94 (81.53, 232.65)	0.101			
		HC	88.13 (56.12, 147.78)	106.56 (69.13, 162.21)	99.52 (64.8, 174.81)	0.047	0.052	0.485	1
		P	<0.001	<0.001	0.016				
	TTL/SA	T2DM	1.42 (1.06, 2.17)	1.38 (1.03, 2)	1.49 (0.96, 2.18)	0.567			
		HC	1.49 (1.1, 2.16)	1.49 (1.11, 2.01)	1.54 (1.09, 2.15)	0.687			
		P	0.497	0.27	0.93				
	MSL-X	T2DM	11.8 (8.75, 15.78)	12.64 (9.35, 16.11)	11.79 (8.26, 16.63)	0.339			
		HC	9.99 (7.5, 13.23)	10.58 (8.04, 14.33)	9.66 (7.24, 14.38)	0.125			
		P	<0.001	<0.001	0.013				
	MSL-Y	T2DM	17.05 (13.62, 21.49)	18.05 (14.45, 22.56)	19.26 (14.44, 23.03)	0.055			
		HC	14.19 (11.65, 18.08)	15.51 (12.03, 19.13)	17.08 (12.99, 19.87)	0.018	0.175	0.028	0.549
		P	<0.001	<0.001	0.028				
	V-X	T2DM	3.4 (2.5, 4.6)	3.7 (2.7, 5)	3.65 (2.85, 5.25)	0.084			
		HC	2.5 (1.9, 3.1)	2.6 (2, 3.4)	2.6 (1.9, 3.5)	0.159			
		P	<0.001	<0.001	<0.001				
	V-Y	T2DM	4.5 (3.65, 6.1)	5 (4.2, 6.6)	5.4 (4.1, 7)	<0.001	0.002	0.005	1
		HC	3.4 (2.8, 4.2)	3.7 (3, 4.8)	3.7 (3.2, 5.3)	<0.001	0.001	<0.001	0.503
		P	<0.001	<0.001	<0.001				
Eyes-closed	TTL	T2DM	266.09 (204.11, 339.98)	294.76 (230.39, 390.78)	316.99 (243.89, 398.45)	<0.001	0.001	<0.001	0.336
		HC	156.06 (127.2, 183.21)	169.24 (140.24, 200.41)	186.49 (152.52, 225.96)	<0.001	<0.001	<0.001	0.061
		P	<0.001	<0.001	<0.001				
	SA	T2DM	161.18 (105.71, 265.14)	212.58 (125.38. 337.77)	216.21 (153.42, 359.44)	<0.001	0.001	<0.001	0.661
		HC	75.47 (52.26, 116.9)	98.38 (64.15, 147.2)	94.77 (67.28, 160.5)	<0.001	<0.001	0.001	1
		P	<0.001	<0.001	<0.001				
	TTL/SA	T2DM	1.6 (1.1, 2.29)	1.4 (0.96, 1.97)	1.27 (0.94, 1.81)	0.002	0.007	0.016	1
		HC	2.01 (1.5, 2.71)	1.72 (1.35, 2.35)	1.8 (1.37, 2.3)	0.001	0.001	0.108	1
		P	<0.001	<0.001	<0.001				
	MSL-X	T2DM	11.69 (8.69, 15.6)	13.78 (9.63, 18.18)	14.44 (10.23, 20.49)	<0.001	0.003	0.002	0.732
		HC	8.11 (6.13, 10.96)	9.37 (6.91, 12.65)	8.78 (6.71, 12.1)	0.004	0.003	0.519	1
		P	<0.001	<0.001	<0.001				
	MSL-Y	T2DM	22.07 (17.57, 27.67)	24.73 (19.27, 31.54)	25.04 (20.4, 32.08)	<0.001	<0.001	0.001	1
		HC	14.51 (12.15, 18.02)	16.54 (14.01, 20.93)	18.18 (14.65, 21.01)	<0.001	<0.001	<0.001	0.129
		P	<0.001	<0.001	<0.001				
	V-X	T2DM	3.9 (2.9, 5.6)	4.6 (3.2, 6.3)	4.8 (3.45, 6.55)	0.003	0.025	0.013	0.826
		HC	2.3 (1.8, 2.9)	2.5 (1.9, 3.1)	2.3 (1.9, 3.3)	0.027	0.024	0.648	1
		P	<0.001	<0.001	<0.001				
	V-Y	T2DM	7.1 (5.4, 9.15)	7.8 (6.3, 10.3)	8.6 (6.7, 10.85)	<0.001	0.001	<0.001	0.234
		HC	4.2 (3.5, 4.9)	4.5 (3.8, 5.5)	5.3 (4.3, 6.3)	<0.001	<0.001	<0.001	0.002
		P	<0.001	<0.001	<0.001				
RQ		T2DM	131.5 (80.9, 207.25)	147.7 (93.1, 228.6)	178.35 (92.3, 274.95)	0.007	0.128	0.01	0.333
		HC	87.55 (54.3, 133.7)	90.1 (62.5, 147.9)	103.6 (73.9, 147.4)	0.026	0.150	0.05	0.838
		P	<0.001	<0.001	<0.001				

T2DM, type 2 diabetes mellitus; HC, healthy control; BMI, body mass index; TTL, total track length; SA, sway area; TTL/SA, track length per unit area; MSL-X, Maximum sway length of CoP along X direction; MSL-Y, Maximum sway length of CoP along Y direction; V-X, velocity of CoP displacements in X direction; V-Y, (velocity of CoP displacements in Y direction; RQ, Romberg quotient; 1 vs 2, Normal weight vs Overweight; 1 vs 3, Normal weight vs Obesity; 2 vs 3, Overweight vs Obesity.

Regarding the static postural control parameters under eyes-closed condition, the statistical analysis results indicated that the T2DM group performed poorly than the HC groups for TTL, SA, TTL/SA, MSL-X, MSL-Y, V-X, and V-Y in all three groups based on BMI category (normal weight, overweight, and obese). For the T2DM group, we found significant difference for TTL, SA, TTL/SA, MSL-X, MSL-Y, and V-X in normal weight participants compared with overweight/obese participants. For the HC group, we also found significant difference for TTL, SA, MSL-Y and V-Y in normal weight participants compared with overweight/obese participants. Interestingly, normal weight participants in the HC group presented significant difference for TTL/SA, MSL-X, and V-X compared with overweight rather than obese participants.

Regarding RQ, significant difference was found between the T2DM and the HC groups in all three groups based on BMI category. Only normal weight participants in the T2DM group presented significant difference for RQ compared with obese participants.

**Table 3** provides the results of generalized linear model between BMI and static postural control parameters while controlling for heart rate, hypertension status, and fasting blood glucose in both T2DM individuals and healthy controls. We found statistically significant correlations between BMI and V-Y under eyes-open condition in both T2DM and healthy control groups (both *P <*0.05). There were also statistically significant correlations between BMI and most static postural control parameters under eyes-closed condition in both groups (all *P <*0.05). RQ was only significantly positive related with BMI in healthy controls (*P* = 0.011).

**Table 3:** Generalized linear model results about the relationship between BMI and static postural control parameters.

		BMI (T2DM group)			BMI (HC group)		
		B	Standard error	*P*	B	Standard error	*p*
Eyes-open	TTL	1.759	1.213	0.147	1.85	0.825	0.025
	SA	2.691	1.907	0.158	−0.422	2.336	0.857
	TTL/SA	0.387	0.297	0.192	0.002	0.011	0.857
	MSL-X	0.102	0.089	0.25	−0.06	0.114	0.596
	MSL-Y	0.174	0.093	0.06	0.165	0.085	0.052
	V-X	0.006	0.027	0.834	0.011	0.018	0.533
	V-Y	0.069	0.028	0.016	0.064	0.02	0.001
Eyes-closed	TTL	5.085	1.734	0.003	3.906	0.991	<0.001
	SA	9.015	3.028	0.003	4.245	1.135	<0.001
	TTL/SA	−0.033	0.016	0.036	−0.034	0.014	0.012
	MSL-X	0.279	0.096	0.004	0.11	0.061	0.07
	MSL-Y	0.537	0.122	<0.001	0.351	0.075	<0.001
	V-X	0.054	0.032	0.093	0.038	0.019	0.041
	V-Y	0.16	0.046	0.001	0.119	0.026	<0.001
RQ		3.622	2.129	0.089	2.671	1.045	0.011

T2DM, type 2 diabetes mellitus; HC, healthy control; BMI, body mass index; TTL, total track length; SA, sway area; TTL/SA, track length per unit area; MSL-X, Maximum sway length of CoP along X direction; MSL-Y, Maximum sway length of CoP along Y direction; V-X, velocity of CoP displacements in X direction; V-Y, velocity of CoP displacements in Y direction; RQ, Romberg quotient.

## Discussion

This study intended to provide new knowledge of static postural control parameter differences between the T2DM and the healthy control participants based on BMI category, and the relationship between BMI and static postural control parameters in the T2DM and the healthy control groups. The present study revealed that the T2DM participants had greater impairment of static postural control compared with the healthy controls in all BMI categories. Normal weight participants had better static postural control for the total track length and the velocity of CoP in medial–lateral direction under eyes-open condition compare with overweight/obese participants in both T2DM and healthy control groups. Normal weight participants also had better static postural control for all related parameters under eyes-closed condition compare with overweight/obese participants in both groups. The correlations between BMI and most static postural control parameters were statistically significant, higher BMI was related with worse static stance control in both T2DM and healthy controls.

Our study found that the T2DM participants presented poorer static postural control than the normal control participants, regardless of BMI categories. The difference between the T2DM and the healthy participants in static postural control are reported in previous researches. Fukunaga (24) evaluated the postural balance (limit of stability, pressure center displacement area, and sway velocity) of 20 T2DM individuals compared with 22 controls using balance posturegraphy equipment. The result found that participants with T2DM had significantly lower limits of stability, higher pressure displacement area, and higher sway velocity on a firm surface with eyes-open/eyes-closed (24), which was in line with our study results. Similarly, another research adopting static posturography showed standing on firm surface under eyes-closed condition had a larger effect in participants with a BMI ≥30 compared to those with a BMI <30 (10). In addition, a recent study by Stolarczyk (25) assessing the fall risk, the general stability, the frontal–posterior index and medial–lateral index between the T2DM and the healthy participants who were homogenic in terms of age and BMI, found a decreased balance and motor coordination in patients with T2DM. Stolarczyk (25) also observed that the postural instability and risk of falling increased with the increase in BMI. The reasons causing impaired static balance function maybe multivariable. Cognitive, proprioceptive (sensory and motor), muscular strength or motor coordination impairment could result in postural control deficits. Firstly, cognitive load plays an important role in the ability to perform basic motor tasks or activities. A cross-sectional study demonstrated that systemic deficit beyond tactile dysfunction and increased BMI contribute to impaired motor function in T2DM, where both working memory functions and balance control were simultaneously impaired in the T2DM group compared to controls (16). Secondly, patients with T2DM had a stiffer plantar soft tissue and thicker Achilles tendon compared with the healthy controls. The changes in biomechanical properties of the ankle–foot complex were correlated with vestibular, somatosensory, and visual inputs to maintain postural stability in T2DM individuals (26). Khan (11) found that T2DM individuals had experienced more falls than healthy controls, a higher postural instability index in neutral position, and decreased motor function including the 6-minute walking test and 5 time sit to stand test. Additionally, the individuals with diabetes demonstrated decreased isokinetic muscle strength of all joints (11). Thirdly, diabetic peripheral neuropathy was a common complication of T2DM, which had a negative impact on the postural control. Peripheral neuropathy leads to the sensory input mechanical receptor damages and adjusted output movement impairment, resulting in poor static balance performance. Although postural control strategy was influenced and correlated with the extent of peripheral neuropathy in T2DM patients (27, 28), Fulk suggested that peripheral neuropathy was not the only cause of impaired balance for T2DM (9). Vaz found that T2DM with or without peripheral neuropathy showed greater anterior–posterior displacement in the unstable platform with eyes-closed compared to the same age healthy individuals (12). No significant difference between the T2DM and the healthy control groups was observed in terms of antero-posterior and medial–lateral displacement in standing firm plate with or without vision (12).

Several risk factors, namely, obesity and impaired postural control were considered to increase the risk of falling and fractures (29). Higher BMI categories were associated with worse static postural control performance in our study. *Post-hoc* pair-wise analysis found that normal weight participants also had better static postural control for most parameters under eyes-open and eyes-closed conditions compared with overweight/obese participants in both T2DM and healthy controls. There were no significant difference on most static balance measures between overweight individuals and obese individuals.

Many previous researches held the same opinion with our findings. A study aiming to determine the body weight contribution to predict balance stability reveal that body weight account for 52% of the variance of balance stability with vision and 54% of the variance without vision (13). A strong correlation between a decreased balance stability and an increased body weight suggested that body weight maybe an emphatic risk factor for falling (13). Another previous study showed that slightly obese participants had an increased mean amplitude and velocity of CoP in anterior–posterior direction compared to normal individuals during quit standing test with eyes-open (30). There are also some possible reasons that may be helpful to clarify these results. Usually, increased amount of abdominal fat is correlated with a higher BMI (31). Consequently, the center of mass is moving forward with respect to the ankle joint in obese individuals, resulting in impaired postural control (17). Evidence showed that obese subjects had an increase of the peak pressure on fore-foot and plantar ground contact area (32). A lower plantar sensitivity due to the hyperactivation of the plantar mechanoreceptors was observed (13). The diminished sensitivity derives from continuous heavier pressure by supporting a larger mass, leading to a poor proprioception input and postural control impairment (33). Meng’s findings indicated that higher BMI was associated with both cognitive function and postural control declines (18). Maktouf evaluated static postural control by CoP displacements during quiet standing, resulting with higher CoP displacements in obese individuals than in controls (34). Electromyography activity data demonstrated that obesity increased the soleus and tibialis anterior muscle coactivation at the ankle joint during static control (34). It was an adaptive neuromuscular response for improving stability by a joint stiffening strategy, which could not be considered as appropriate adaptation (34). Another study of Maktouf concluded that decreased fatigability threshold of plantar and dorsal flexors could partly contribute to postural control alterations in obese individuals (35).

The present study demonstrated no significant differences between normal weight individuals and overweight/obese individuals in SA, TTL/SA, MSL-X, and V-X under eyes-open condition for both T2DM and healthy controls. However, there was a greater number of association between the postural control variables and the BMI in the the eyes-closed condition than in the eyes-open condition for both T2DM and control groups. There were statistically significant differences of these parameters under eye-closed condition, which revealed that vision was of importance for static balance, especially for high BMI individuals. A previous cross-sectional study which was to assess the obesity affecting balance control in older women, showed that the obese participants had an decreased postural stability because of increasing CoP speed (33). The study by Dutil (33) demonstrated a significant interaction effect between group and vision conditions, revealing that higher BMI group was more affected by no-vision condition than normal weight group, which was in line with our research result.

These results supported that a sensory component was important for balance control in maintaining stability. It was well known that once visual sensory component is removed during maintaining static stance, other sensory components (proprioception) acted on a greater role to compensate and maintain postural stability (36). Obesity was related to a decrease in postural stability when vision was not available compared to normal weight and overweight by previous research, which suggests that obese participants should be more dependent on vision to control balance (37). There were also significant differences in Romberg quotient between the T2DM and the healthy controls, showing that T2DM individuals maybe dependent on vision to maintain static balance. Additionally, there were significant differences in Romberg quotient between normal weight and obese T2DM individuals, whereas no significant difference among all BMI categories healthy controls. These findings mean that obese T2DM individuals rely more on vision to maintain static postural balance.

Our finding had important implications for clinical and research aspects. T2DM and higher BMI had negative impact on static postural control. The static postural control performance in eyes-closed condition was worse than that in eyes-open condition for all participants, especially for T2DM patients. The finding suggested that T2DM patients should manage their weight better, and a good visual environment was needed in their work and life so as to reduce the related injuries caused by postural control impairment. A strength of our study was the fact that the T2DM group and the healthy control group were matched according to age, sex, and BMI, where the influence of some potential confounding factors could be mitigated. Another strength was that our study recruited a much larger sample size of participants than previous related research. However, several limitations should be acknowledged. Firstly, we could not clarify the underlying mechanisms for the result that the T2DM group had worse static balance performance than the healthy controls. In addition, some factors affecting the results were not collected such as diabetic peripheral neuropathy, diabetes duration, and physical activity amounts. Secondly, only two-leg static stance on a firm force-plate under eyes-open/eyes-closed conditions was evaluated in our study. Thirdly, the participants of this research were from ages 55 to 75 that involved middle age and elderly people; a future study could focus on a specific age group and develop more on the relationship between this age group and postural impairment. Finally, our study was a cross-sectional designed research that could not consider the causal and effect inference. These limitations should be considered in future research designs to determine the link of BMI and postural control performance in individuals with or without T2DM.

In conclusion, the results of this research suggested that T2DM patients had impaired static postural control performance compared to healthy controls. The results also indicated the association between BMI and static postural balance, where higher BMI individuals showed more static postural instability in both T2DM and healthy controls.

## Data Availability Statement

The raw data supporting the conclusions of this article will be made available by the authors, without undue reservation.

## Ethics Statement

All procedures in this study that involved human participants were performed in accordance with the ethical standards of the Second Affiliated Hospital of Fujian University of Traditional Chinese Medicine Ethics Committee (approval number SPHFJP-K2019059-02). The patients/participants provided their written informed consent to participate in this study.

## Author Contributions

LY and JQ contributed equally to this work and share first authorship. LY and JQ designed the study and wrote the manuscript. YC, JX, and CH selected data. JH and YX did statistical analysis. ZL and JT reviewed the manuscript and contributed to the discussion. All authors contributed to the article and approved the submitted version.

## Funding

This study is supported by the Key Research and Development Project funded by the Ministry of Science and Technology of the People’s Republic of China (grant number: 2019YFC1710301) and the National Science Foundation Project funded by the Science and Technology Department of Fujian Province (grant number: 2019J01481).

## Conflict of Interest

The authors declare that the research was conducted in the absence of any commercial or financial relationships that could be construed as a potential conflict of interest.

## Publisher’s Note

All claims expressed in this article are solely those of the authors and do not necessarily represent those of their affiliated organizations, or those of the publisher, the editors and the reviewers. Any product that may be evaluated in this article, or claim that may be made by its manufacturer, is not guaranteed or endorsed by the publisher.
